# Supplementary material for: Bacterial lysis, autophagy and innate immune responses during adjunctive phage therapy in a child
Source: EMBO Mol Med. 2021 Aug 9;13(9):e13936. doi: 10.15252/emmm.202113936 (PMC8422068; doi:10.15252/emmm.202113936)
Supplement: Supplementary file 2 — Expanded View Figures PDF [file EMMM-13-e13936-s001.pdf]

Expanded View Figures

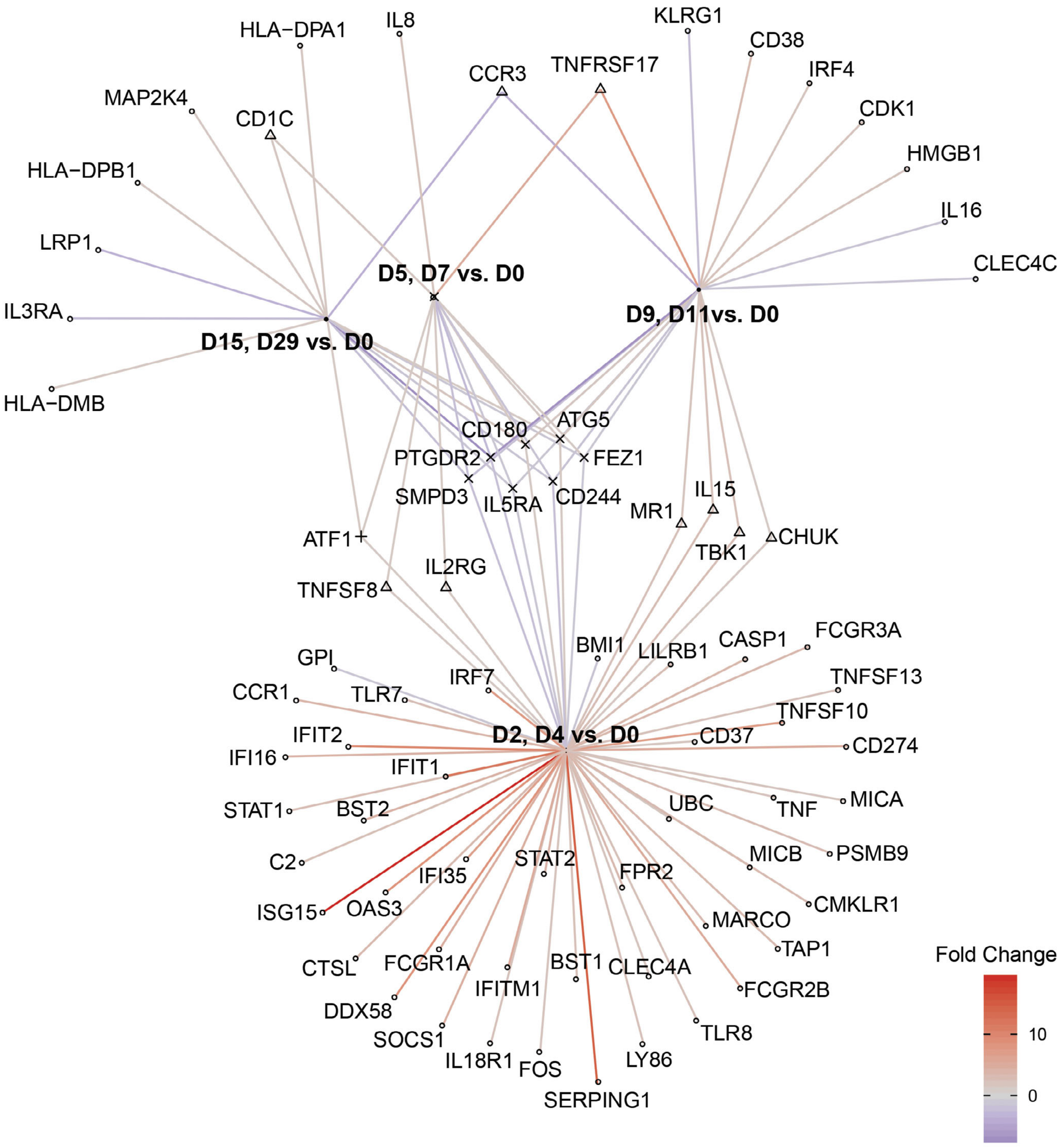

Figure EV1. Gene network visualisation of significant differentially expressed immune response over 2 weeks of intravenous anti-pseudomonal adjunctive phage therapy with 2 weeks of follow-up.

D0: pre-phage, D2–D11: during phage administration, D15: one day post-adjunctive phage therapy, D29: 15 days post-adjunctive phage therapy. The gene network highlights the significant gene expression changes at the beginning of adjunctive phage therapy (D2-4), and the overlaps of common genes for the duration of adjunctive phage therapy.

© 2021 The Authors

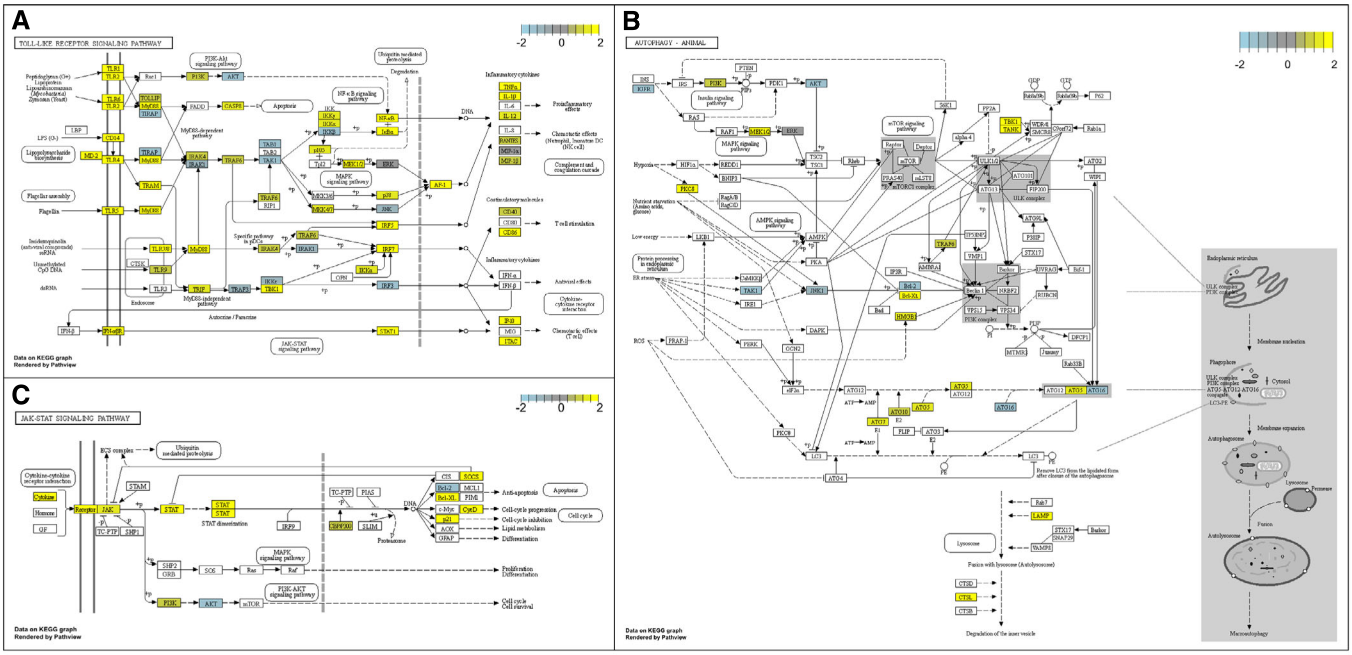

Figure EV2. Differentially expressed genes in major inflammation-associated pathways.

Visualisation using PathView following Gene Set Enrichment Analysis and Pathway enrichment analysis (KEGG, https://www.genome.jp/kegg/); genes with fold change > 2 and < −2 were integrated into KEGG pathways.

A Toll-like receptor signalling pathway.
B Autophagy.
C JAK-STAT signalling pathway.

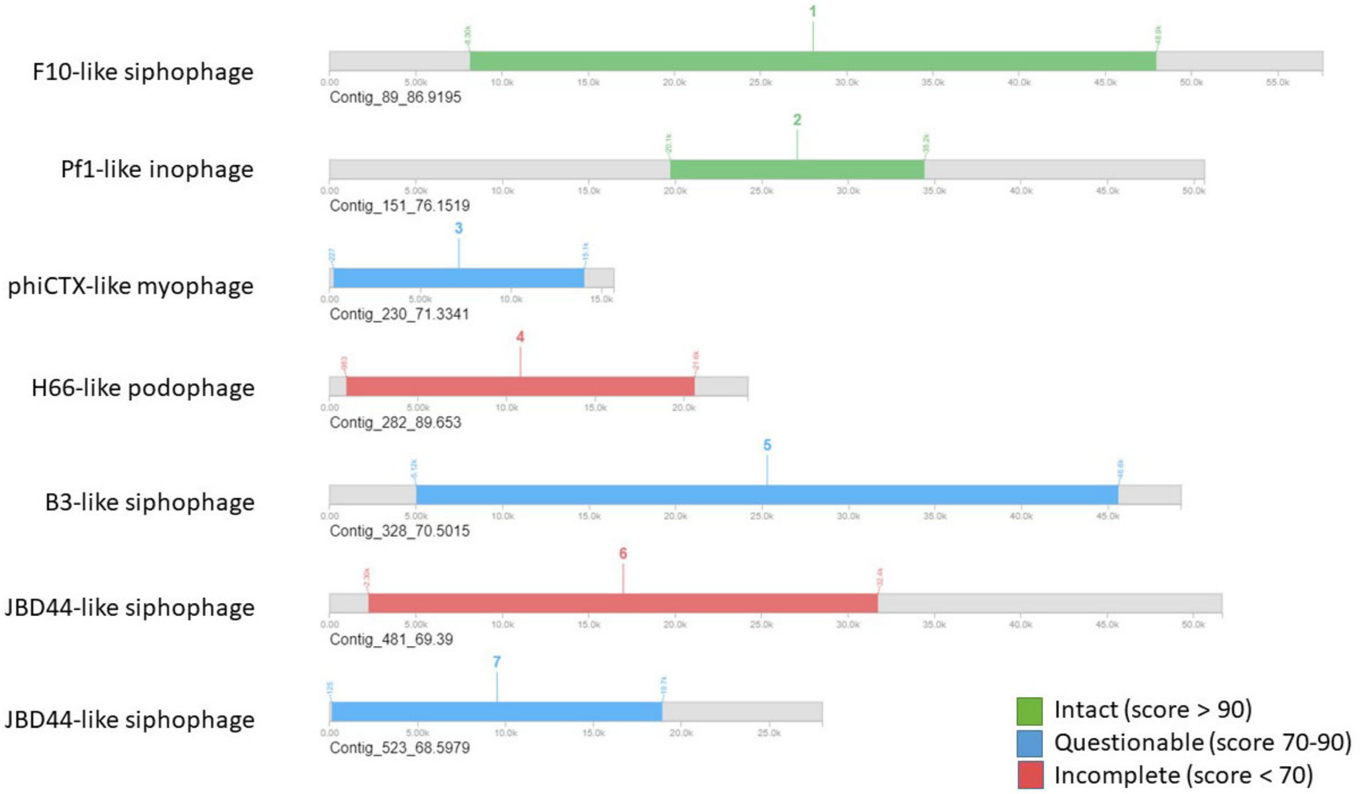

Figure EV3. Prophage sequences from PHAge Search Tool Enhanced Release (PHASTER) for the clinical isolate of *Pseudomonas aeruginosa* (Ppa2.1).

© 2021 The Authors
